# Associations of Polymorphisms Localized in the 3′UTR Regions of the *KRAS*, *NRAS*, *MAPK1* Genes with Laryngeal Squamous Cell Carcinoma

**DOI:** 10.3390/genes12111679

**Published:** 2021-10-23

**Authors:** Ruta Insodaite, Alina Smalinskiene, Vykintas Liutkevicius, Virgilijus Ulozas, Roberta Poceviciute, Arunas Bielevicius, Laimutis Kucinskas

**Affiliations:** 1 Institute of Biology Systems and Genetic Research, Lithuanian University of Health Sciences, 50103 Kaunas, Lithuania; alina.smalinskiene@lsmuni.lt (A.S.); roberta.poceviciute@lsmu.lt (R.P.); arunas.bielevicius@lsmuni.lt (A.B.); laimutis.kucinskas@lsmuni.lt (L.K.); 2 Department of Otorhinolaryngology, Lithuanian University of Health Sciences, 50161 Kaunas, Lithuania; vykintas.liutkevicius@lsmuni.lt (V.L.); virgilijus.ulozas@lsmuni.lt (V.U.)

**Keywords:** laryngeal squamous cell carcinoma, single-nucleotide polymorphisms, mitogen-activated protein kinases, *KRAS*, *NRAS*, *MAPK1*

## Abstract

Background: Genetic variations, localized in the 3′ untranslated region (UTR) in mitogen-activated protein kinase (MAPK) pathway-related genes, may alter the transcription and impact the pathogenesis of laryngeal squamous cell carcinoma (LSCC). The present study investigated the associations of single-nucleotide polymorphisms (SNP), localized in the 3′UTR) of the *KRAS*, *NRAS,* and *MAPK1* genes with LSCC risk and clinicopathological features. Methods: Genomic DNA and clinical data were collected from 327 adult men with LSCC. The control group was formed from 333 healthy men. Genotyping of the SNPs was performed using TaqMan SNP genotyping assays. Five *KRAS*, *NRAS,* and *MAPK1* polymorphisms were analyzed. All studied genotypes were in Hardy–Weinberg equilibrium and had the same allele distribution as the 1000 Genomes project Phase 3 dataset for the European population. Results: Significant associations of the studied SNPs with reduced LSCC risk were observed between *NRAS* rs14804 major genotype CC. Significant associations of the studied SNPs with clinicopathologic variables were also observed between *NRAS* rs14804 minor T allele and advanced tumor stage and positive lymph node status. SNP of *MAPK1* rs9340 was associated with distant metastasis. Moreover, haplotype analysis of two *KRAS* SNPs rs712 and rs7973450 revealed that TG haplotype was associated with positive lymph node status in LSCC patients. Conclusions: According to the present study, 3′UTR SNP in the *NRAS* and *MAPK1* genes may contribute to the identifications of patients at higher risk of LSCC lymph node and distant metastasis development.

## 1. Introduction

Laryngeal squamous cell carcinoma (LSCC) is one of the most common head and neck cancers, accounting for about 2.4% of newly diagnosed cases and 0.7% of all cancer-related deaths occurring worldwide per year [1]. The main problem is late-stage diagnosis: up to 40% of patients present with advanced disease and are associated with significant morbidity and mortality for the patient and increased financial costs for society [2]. The disease is typically diagnosed in male patients, usually between the fifth and seventh decade of life, and is extremely rare in adolescents or children [3,4]. The development of LSCC is multifactorial: the combination of genetic mutations, epigenetic dysregulation, and etiologic factors such as tobacco and alcohol consumption, drives together to promote the initiation and further development of the disease [5,6].

The mitogen-activated protein kinases (MAPK) are the type of proteins that are activated when dually phosphorylated on serine and threonine. MAPK is the terminal or effector kinase in three-kinase phosphorelay cascade in which MAPKs are phosphorylated and activated by mitogen-activated protein kinase kinase (MAPKK), which themselves are phosphorylated and activated by mitogen-activated protein kinase kinase kinase (MAPKKK) [7]. The activation of MAPK cascade occurs as a response to extracellular stimuli, including growth factors, stress such as cytokines, toxins, drugs, changes in cell adherence, osmolarity, temperature, and oxygen radicals [7,8]. The active terminal kinase is transferred to the nucleus, where phosphorylate specific substrate proteins, usually, transcription factors, that regulate cell proliferation, survival, motility, metabolism, transcription, and translation [8,9].

One of three well-known MAPK pathways in mammalian cells is an extracellular signal-regulated kinase (ERK1/2) or MAPK1/2. The classic activation of ERK1 and ERK2 isoforms is initiated by the binding of a ligand to the receptor at the plasma membrane. The receptor transmits an activating signal by recruiting SOS (son of sevenless) to stimulate Rat sarcoma virus (Ras) and convert GTP to GDP. These changes activate Ras and initiate the interaction with Raf a MAPKKK. Active Raf activates the MEK or MAPKK, which, in turn, phosphorylates the ERK1/2 or MAPK. Activated ERK isoforms are transferred to the nucleus or stay in the cytoplasm [10,11]. Due to a wide range of nuclear and cytoplasmic targets, Ras/Raf/MEK/ERK1/2 cascade participates in cell proliferation, differentiation, death and survival, migration, cytoskeletal remodeling processes [9,10,11,12].

Despite a wide range of biological functions, recent studies showed that activation of the Ras/Raf/MEK/ERK1/2 cascade is related to pathogenesis, progression, and oncogenic behavior of human cancer including breast and colorectal cancer, head and neck squamous cell carcinoma [13,14,15,16]. The main factor associated with the Ras/Raf/MEK/ERK1/2 pathway that promotes carcinogenesis is the hyperactivation of this cascade. Hyperactive Ras/Raf/MEK/ERK1/2 signaling exists in over 85% of cancer cases, which are caused by genetic alterations of its upstream activators, especially RAS family protein isoforms (KRAS, NRAS, HRAS) [13]. The association with RAS and LSCC clinicopathological features has been demonstrated by several authors. Overexpression of RAS is linked to aggressive cancer phenotype, recurrence, and poor platinum response [17,18]. In LSCC, the most common are the *NRAS* isoform mutations, which have been detected in 9.7% of all LSCC cases. *KRAS* mutation frequency varies from 4.3% to 11.5%, according to different studies. Mutated *HRAS* isoform is detected in less than 1% of LSCC cases [19,20].

Mutations in effector protease ERK2 or *MAPK1* gene are rare, and increased activity is due to changes in upstream components. However, some studies showed that *MAPK1* mutation causes resistance to Raf/Mek inhibitors due to reduced dual-specificity phosphatase binding and loss of negative regulation in cervical and head and neck carcinoma [21,22]. Point mutations increase *MAPK1* specific activity and reduce sensitivity to Mek inhibitors [23].

Single-nucleotide polymorphisms (SNP) in Ras/Raf/MEK/ERK1/2 cascade genes may play a role in the development and course of LSCC. Five functional SNPs in *KRAS* (rs712, rs61764370, rs7973450), *NRAS* (rs14804), and *MAPK1* (rs9340) that are located in the 3′UTR of mRNAs may have a role in Ras/Raf/MEK/ERK1/2 cascade homeostasis changes due to modifications in miRNAs binding sites [24,25,26,27,28,29]. Case-control studies demonstrated links between *KRAS* and *NRAS* genes polymorphisms and squamous cell carcinomas [30,31,32,33,34]. However, to the best of our knowledge, there are no data in the literature about the possible links between *MAPK1* gene polymorphisms and LSSC development. Links between selected functional SNPs in RAS isoforms genes and LSCC risk and clinicopathological features are also poorly investigated.

Therefore, the aim of the present study was to investigate the contribution of *KRAS*, *NRAS,* and *MAPK1* gene functional polymorphisms to the development and clinicopathologic features of LSCC.

## 2. Materials and Methods

### 2.1. Study Population

The study group consisted of 327 adult Lithuanian men with LSCC. All patients were treated in the Department of Otorhinolaryngology of the Lithuanian University of Health Sciences (LUHS), Kaunas, Lithuania. A detailed otorhinolaryngological examination (flexible endoscopy and/or video laryngostroboscopy, neck palpation, direct microlaryngoscopy with biopsy) was performed for all LSCC patients. The diagnosis of LSCC was histologically confirmed at the Department of Pathology, LUHS. The final diagnosis of LSCC was based on clinical data and the results of histological examination and laryngeal and neck CT or MRT. The exclusion criteria were other malignancies and significant comorbidities. The characteristics of pathological features were obtained for all patients from pathologist records.

The reference group consisted of 333 healthy Kaunas city male residents who were selected during the international Health, Alcohol and Psychosocial Factors in Eastern Europe project (HAPPIE) study [35]. The sample was collected by the Population Study Laboratory at the Institute of Cardiology of LUHS by carrying out a selection based on the lists of residents of Kaunas city. The exclusion criteria were malignancies and significant comorbidities, female gender, age outside the patient sample variation range.

The present study was approved by the Kaunas Regional Biomedical Research Ethics Committee (Protocol No. BE-2-37) and informed consents were obtained from all the participants prior to inclusion in the study.

### 2.2. Candidate Polymorphisms

The genes and polymorphisms known to modulate Ras/Raf/MEK/ERK1/2 cascade were selected. The selection criteria included: (i) functional SNPs in the *KRAS*, *NRAS,* and *MAPK1* genes located in the 3′UTR regions, where miRNA binds; (ii) SNPs relevant to outcomes in other settings; and (iii) SNPs with a minor allele frequency greater than 10% in the study population. We selected five functional SNPs: the *KRAS* gene rs712, rs61764370, and rs7973450; the *NRAS* gene rs14804; and the *MAPK1* gene rs9340.

### 2.3. DNA Extraction and Genotyping

Peripheral blood samples from the study population were collected in ethylenediaminetetra-acetic acid tubes. Genomic DNA from peripheral blood leukocytes was extracted with a DNA extraction kit (Thermo Fisher Scientific Baltics, Vilnius, Lithuania), according to the manufacturer’s recommendations.

Genotyping of the selected SNPs was performed at the Dr. K. Janusauskas Laboratory of Genetics of the Institute of Biology Systems and Genetic Research of LUHS. The polymorphisms of the target genes were estimated by using commercially available TaqMan Genotyping kits (C_189219680_10, rs61764370, C_189571552_10, C___8701397_10, C___7626904_10; Applied Biosystems; Thermo Fisher Scientific, Inc.). The Applied Biosystems 7900HT Real-Time Polymerase Chain Reaction system (Applied Biosystems; Thermo Fisher Scientific, Inc., Waltham, MA, USA) was employed for SNPs detection. The cycling program was initiated by heating up to 95 °C for 10 min followed by 40 cycles (at 95 °C for 15 s and at 60 °C for 1 min). Finally, allelic discrimination was performed by using the SDS 2.3 software (Applied Biosystems; Thermo Fisher Scientific, Inc., Waltham, MA, USA). For negative control, nuclease-free ddH_2_O was used, while for positive control, the DNA of the known genotype was used. Each sample genotyping was repeated twice for accuracy.

### 2.4. Statistical Analysis

The allele frequency distributions of the investigated SNPs were compared with the European population data from the 1000 Genomes project phase 3 database [36]. For each SNP, a Hardy–Weinberg equilibrium was assessed by using Pearson’s Chi-square and Fisher’s exact tests. The association between the *KRAS*, *NRAS,* and *MAPK1* polymorphisms and LSCC was estimated by computing the odds ratios (ORs) and then 95% confidence intervals (CI) from logistic regression in genotype and allelic models. The Haploview v4.1 software was used to check for the linkage disequilibrium between KRAS SNPs [37]. The associations of SNPs with LSCC clinicopathologic variables such as tumor stage, lymph node status, distant metastasis, tumor grade, were evaluated by Pearson’s Chi-square or Fisher’s exact test. ORs and their 95% CI, from logistic regression in genotype and allelic models, were recorded for each tested marker. Results were statistically significant when *p* was less than 0.05. Statistical analysis was performed using SPSS for Windows v20.0 software (Released 2011; IBM Corp, Armonk, NY, USA).

## 3. Results

### 3.1. Sample Characteristics

A total of 327 Lithuanian male patients with LSCC were included in the current analysis. The frequency of tumor clinical factors is shown in Table 1. All 333 persons from the control group were also included. The LSCC patients and reference groups were matched by age (*p* > 0.05) (Table 2) and gender.

All subjects were genotyped for a panel of five SNPs: The *KRAS* gene rs712, rs61764370, and rs7973450; the *NRAS* gene rs14804; and the *MAPK1* gene rs9340. The genotypes were found to be in Hardy–Weinberg equilibrium in the five SNPs. Linkage disequilibrium between two *KRAS* polymorphisms was confirmed (R^2^ = 0.35). The cohort of the present study manifested a similar allele distribution to that of the European population that was analyzed in the 1000 Genomes project phase 3 (Table 3).

### 3.2. Case-Control Analysis

The distributions of *KRAS*, *NRAS,* and *MAPK1* SNPs genotypes and alleles in LSCC and control groups are presented in Table 4. We observed that, in the LSCC group, minor allele T frequency of *NRAS* rs14804 is statistically significantly lower, compared to the reference group (24.6 vs. 30.0; *p* = 0.031). Associations between analyzed *KRAS* gene rs712, rs61764370, and rs7973450; the *NRAS* gene rs14804; and the *MAPK1* gene rs9340 SNPs and LSCC according to genotypes and allelic models are presented in Table 5. Analysis showed that *NRAS* polymorphism rs14804 wild-type genotype CC reduces the risk of LSCC development (OR 0.754; 95% CI: 0.754–0.925; *p* = 0.047).

### 3.3. Associations of SNPs with Clinicopathological Features of LSCC

The analysis results on associations between the analyzed SNPs and clinicopathologic features of LSCC are shown in Tables S1–S5. A significant link between the *NRAS* rs14804 wild-type CC genotype and the early tumor stage (OR 0.215; CI: 0.061–0.757; *p* = 0.017) was revealed in the single-locus analysis.

The allelic model showed that the minor allele T of this SNP is associated with the advanced cancer stage (OR 4.656; 95% CI: 1.321–16.407; *p* = 0.017). Patients carrying the *NRAS* rs14804 T allele were also predisposed to positive lymph node status (OR 3.646; 95% CI: 2.334–9.249; *p* = 0.023). Additionally, *MAPK1* polymorphism rs9340, in the genotype model, was linked to distant metastasis. Specifically, 14.8% of LSCC patients carrying the *MAPK1* rs9340 TT genotype had distant metastasis, compared to 0.7% noncarriers (OR 4.553; 95% CI: 1.069–8.461; *p* = 0.041).

Other analyzed polymorphisms of *KRAS* in neither genotype nor allelic models revealed no associations with the clinicopathological factors of LSCC (Tables S1–S5).

Linkage disequilibrium analysis showed that two *KRAS* SNPs are in disequilibrium SNPs (R^2^ = 0.35), meaning that the associations were not independent. According to that, haplotype analysis was performed to explore the relationship between two *KRAS* polymorphisms and LSCC clinicopathological features. Phasing revealed three possible *KRAS* (rs712 and rs7973450) haplotypes GT (52%), TT (26%), and TG (22%). The TG haplotype was further related to positive lymph node status (OR 3.954; 95% CI 2.577–6.578; *p* = 0.015; Table S6).

## 4. Discussion

In the present study, associations between five functional SNPs in three Ras/Raf/MEK/ERK1/2 cascade genes (i.e., *KRAS*, *NRAS*, and *MAPK1*) and the risk of LSCC development were investigated. Also, associations between the analyzed SNPs and clinicopathologic profiles in a cohort of LSCC patients were analyzed. We found that *NRAS* SNP rs14804 minor allele (T) frequency is higher in the control group compared to the LSCC group. Also, CC genotype was associated with decreased risk of the disease. Furthermore, we confirmed that the T allele relates to the advanced LSCC stage and positive lymph node status. So far, only Jin et al. have analyzed *NRAS* SNP associations with malignant disease [28]. However, no significant associations with carcinoma were detected in that study.

A study by Jiang et al. found that SNPs localized in the 3′UTR of the *KRAS* affected cancer risk through altering the activation of the gene [38]. Huang et al. showed that the *KRAS* rs712 T allele is significantly associated with metastasis and may predict a poor clinical outcome in colorectal cancer [39]. Similar results were presented in a case-control study of breast and gastric squamous cell carcinomas [40,41]. Interesting results were presented in a Papanikolaou et al. study, where overexpression of KRAS was observed in all grades of the examined primary LSCC tissue [17]. However, our findings do not suggest that *KRAS* rs712 polymorphism is associated with LSCC development and clinicopathological features. Comparing the insights mentioned above with our results, we should consider conducting a new study based on LSCC tumor samples analysis. Based on the study results of Papanikolaou et al., we should combine *KRAS* rs712 polymorphism study with immunohistochemistry assay to determine the possible association of *KRAS* functional polymorphism with changes in protein expression level.

Another analyzed *KRAS* SNP localized in the 3′UTR region found no significant associations with LSCC risk and clinicopathological features too. Santiago et al. analyzed 165 males diagnosed with squamous cell carcinoma of the head and neck (HNSCC), and 230 healthy male subjects without cancer and a family history of cancer; results found that *KRAS* rs61764370 has no association with disease [42]. Contrasting data provided by Weidhaas et al., who analyzed 116 DNA samples from HNSCC patients, found that rs61764370 is a potentially promising biomarker of poor prognosis [18]. Due to the fact that LSCC assigned to the HNSCC group and that analysis for blood samples may not reflect mutations in tumor tissue, we should also consider a new study based on tumor sample analysis to specify these contrasting data.

In the present study, the third *KRAS* rs7973450 also did not show any associations with LSCC development and clinicopathological features. In general, we were unable to find any studies analyzing the associations of this polymorphism with HNSCC. However, some studies showed that the rs7973450 mutant GG genotype is associated with significantly increased neuroblastoma and hepatoblastoma risk [43,44].

It is important to mention that, in the present study, the haplotype analysis of two *KRAS* SNPs rs712 and rs7973450 revealed a significant association between minor alleles TG haplotype and lymph node status of LSCC patients (OR 3.954; 95% CI 2.577–6.578; *p* = 0.015) (Table S6). This haplotype in Nigh et al.’s study was linked to increased thyroid carcinoma risk in the Chinese population; however, TG haplotype associations with disease clinicopathological features were not analyzed [45].

Associations of *MAPK1* polymorphism rs9340 with malignant diseases were analyzed in several studies. Heyde et al. reported that there is no association between minor T allele and human epidermal growth factor receptor-2 positive breast cancer [46]. Similar data are proved by Gonzalez-Hormazabal et al.’s study, where the associations between *MAPK1* polymorphisms and gastric cancer were analyzed. Although experimental data suggest that *MAPK1* rs9340 does not play a role in malignant diseases, our findings revealed that mutant TT genotype is associated with distant metastasis in LSCC.

To summarize our results, first, we should mention that five functional SNPs, selected for this study, are localized in 3′UTR of the gene, where the miRNA binds directly. This binding then induces transcript cleavage or translational repression depending on the level of complementarity between the miRNA and mRNA transcript. Polymorphisms in this region may affect miRNA regulation, either by disrupting or creating a miRNA binding site, and cause transcription changes [47]. Our results revealed that *NRAS* and *MAPK1* polymorphisms are associated with clinicopathological features of LSCC. These findings may consider the speculation that these SNPs change miRNA and mRNA binding sites complementary, causing increased transcription activity and Ras/Raf/MEK/ERK1/2 cascade hyperactivation, which promotes carcinogenesis, according to the literature [24,25,26,27,28,29]. According to these speculations, in future studies, it would be appropriate to combine SNP, miRNA, and protein expressions research to identify possible correlations between the genotype and phenotype. As mentioned above, a potential limitation of our study is that *MAPK1*, *KRAS*, and *NRAS* SNPs were analyzed in the blood and not in tumor samples. Potentially, the observation of these polymorphisms in tumor tissue would prove a significant association between *KRAS* rs712, rs61764370 and rs7973450 SNPs and LSCC clinicopathological features. We also acknowledge that *KRAS*, *NRAS*, and *MAPK1* measurements were not available in the current study. However, the results of our study support the relevance of Ras/Raf/MEK/ERK1/2 cascade gene polymorphisms to malignant diseases.

## 5. Conclusions

In conclusion, the results of the present study proved an association between the *NRAS* rs14804 minor T allele and advanced LSCC tumor stage. Furthermore, associations of the *MAPK1* rs9340 genotype TT with LSCC distant metastasis were revealed. Additionally, haplotype analysis of *KRAS* rs712 and rs7973450 showed that TG haplotype is associated with LSCC metastasis to lymph nodes. Our findings confirm the presumption that functional SNPs in the 3′UTR regions of Ras/Raf/MEK/ERK1/2 cascade genes are associated with LSCC development and clinicopathological features.

## Figures and Tables

**Table 1:** Frequency data for patients clinicopathological factors.

Factor	Patients, *n* (%)
Age, years	
<60 years	107 (32.7)
≥60 years	220 (67.3)
Tumor stage	
T1 and T2	175 (53.5)
T3 and T4	152 (46.5)
Lymph node status	
Positive	86 (26.3)
Negative	241 (73.3)
Distant metastasis	
Positive	10 (3.1)
Negative	317 (96.9)
Grade	
G1 and G2	287 (88.3)
G3	38 (11.7)

**Table 2:** Characteristics of study groups.

Characteristic	Mean	Standard Deviation	*p*-Value
Age, years			
LSCC patients (*n* = 327)	62.96	8.58	0.075 ^1^
Control group (*n* = 333)	63.64	7.89	

^1^ Student’s *t*-test, for independent samples. LSCC—laryngeal squamous cell carcinoma.

**Table 3:** Allele frequencies of analyzed single nucleotide polymorphisms in the study population and European population data from the 1000 Genomes Project Phase 3 database.

Gene	SNP	Major Allele Frequency		Minor Allele Frequency	
		Study Allele Frequencies	1000 Genomes Project Phase 3 Database Allele Frequencies	Study Allele Frequencies	1000 Genomes Project Phase 3 Database Allele Frequencies
*KRAS*	rs712 G > T	0.58	0.52	0.42	0.48
	rs61764370 T > G	0.90	0.90	0.10	0.10
	rs7973450 T > G	0.76	0.81	0.24	0.22
*NRAS*	rs14804 C > T	0.73	0.75	0.27	0.25
*MAPK1*	rs9340 C > T	0.57	0.56	0.43	0.44

**Table 4:** Distribution of genotypes and alleles frequencies for polymorphisms at *KRAS*, *NRAS*, *MAPK1* genes in LSCC (*n* = 327) and control (*n* = 333) groups.

Gene	SNP; Position	Group	Genotype Frequency			*p*-Value	MAF	*p*-Value
*KRAS*	rs712		GG	GT	TT	0.746	T	0.954
	G > T	LSCC	33.3	48.3	18.3		42.5	
	Chr.12:25209618	Control	35.1	45.3	19.5		43.2	
	rs61764370		TT	TG	GG	0.583	G	0.931
	T > G	LSCC	86.9	13.1	0		6.5	
	Chr.12:25207290	Control	87.4	12.3	0.3		6.4	
	rs7973450		TT	TG	GG	0.801	G	0.944
	T > G	LSCC	58.4	34.9	6.7		24.2	
	Chr.12:25207290	Control	59.2	33	7.8		24.3	
*NRAS*	rs14804		CC	CT	TT	0.103	T	**0.031** ^1^
	C > T	LSCC	56.3	38.2	5.5		24.6	
	Chr.1:114707222;	Control	49.2	42	8.7		30	
*MAPK1*	rs9340		CC	CT	TT	0.701	T	0.408
	C > T	LSCC	33.3	50.2	16.5		41.6	
	Chr.22:21761064	Control	30.6	51.1	18.3		43.8	

^1^ Significant difference. MAF: minor allele frequency. LSCC: laryngeal squamous cell carcinoma.

**Table 5:** Association between *KRAS*, *NRAS*, *MAPK1* polymorphisms in allele and genotype models and LSCC development.

Gene	SNP	Model	OR	95% CI	*p*-Value
*MAPK1*	rs9340	Genotype model:			
		CC vs. CT and TT	0.883	0.637–1.225	0.883
		TT vs. CT and CC	1.134	0.758–1.698	0.541
		CT vs. CC and TT	1.037	0.764–1.407	0.818
		Allelic model:			
		C carrier vs. C noncarrier	0.882	0.590–1.320	0.541
		T carrier vs. T noncarrier	1.132	0.816–1.571	0.457
*KRAS*	rs712	Genotype model:			
		GG vs. GT and TT	1.083	0.785–1.494	0.626
		TT vs. GT and GG	1.079	0.731–1.594	0.701
		GT vs. GG and TT	0.887	0.654–1.205	0.444
		Allelic model:			
		G carrier vs. G noncarrier	0.927	0.628–1.368	0.701
		T carrier vs. T noncarrier	0.923	0.669–1.273	0.626
	rs61764370	Genotype model:			
		TT vs. TG and GG	1.049	0.665–1.654	0.837
		GG vs. TG and TT	-	-	-
		TG vs. TT and GG	0.927	0.587–1.466	0.747
		Allelic model:			
		G carrier vs. G noncarrier	0.953	0.604–1.503	0.837
		T carrier vs. T noncarrier	-	-	-
	rs7973450	Genotype model:			
		TT vs. TG and GG	1.031	0.756–1.406	0.845
		GG vs. TG and TT	1.174	0.651–2.117	0.594
		TG vs. TT and GG	0.922	0.668–1.272	0.62
		Allelic model:			
		G carrier vs. G noncarrier	0.97	0.711–1.322	0.845
		T carrier vs. T noncarrier	0.852	0.472–1.534	0.594
*NRAS*	rs14804	Genotype model:			
		CC vs. CT and TT	0.754	0.555–0.925	**0.047** ^1^
		TT vs. CT and CC	1.637	0.891–3.011	0.112
		CT vs. CC and TT	1.172	0.858–1.601	0.318
		Allelic model:			
		C carrier vs. C noncarrier	0.611	0.332–1.123	0.112
		T carrier vs. T noncarrier	1.326	0.976–1.801	0.071

^1^ Significant difference. OR: Odds ratio. CI: confidence intervals. LSCC: laryngeal squamous cell carcinoma.

## Data Availability

Not applicable.

## Supplementary Materials

The following are available online at https://www.mdpi.com/article/10.3390/genes12111679/s1, Table S1: Associations between *KRAS* rs712 polymorphism and LSCC clinicopathological features, Table S2: Associations between *KRAS* rs61764370 polymorphism and LSCC clinicopathological features, Table S3: Associations between *KRAS* rs7973450 polymorphism and LSCC clinicopathological features, Table S4: Associations between *NRAS* rs14804 polymorphism and clinicopathological features, Table S5: Associations between *MAPK1* rs9340 polymorphism and LSSC clinicopathological features, Table S6: Association between *KRAS* rs712 and rs7973450 polymorphisms in haplotype models and LSCC clinicopathologic features.
