# Supplementary material for: Associations of Polymorphisms Localized in the 3′UTR Regions of the KRAS, NRAS, MAPK1 Genes with Laryngeal Squamous Cell Carcinoma
Source: Genes (Basel). 2021 Oct 23;12(11):1679. doi: 10.3390/genes12111679 (PMC8625477; doi:10.3390/genes12111679)
Supplement: Supplementary file 1 [file genes-12-01679-s001.zip › genes-1401549-supplementary.pdf]

Table S1. Associations between *KRas* rs712 polymorphism and LSCC clinicopathological features.

**Parameter**	**Genotype model**			**Allelic model**	
	**GG vs. GT and TT**	**TT vs. GT and GG**	**GT vs. GG and TT**	**G carrier vs. G noncarrier**	**T carrier vs. T noncarrier**
Tumor stage					
T1 and T2	52.3 % vs. 54.1 %	46.7 % vs. 55.1 %	57.0 % vs. 50.3 %	55.1 % vs. 46.7 %	54.1 % vs. 52.3%
T3 and T4	47.7 % vs. 45.7 %	53.3 % vs. 44.9 %	43.0 % vs. 49.7 %	44.9 % vs. 53.3 %	45.1 % vs. 47.7 %
OR (95% CI)	1.076 (0.679-1.706)	1.400 (0.798-2.455)	0.765 (0.494-1.182)	1.400 (0.798-2.455)	0.929 (0.586-1.473)
P-value	0.754	0.240	0.227	0.240	0.754
Lymph node status					
Positive	30.3 % vs. 24.3%	28.3 % vs. 25.8 %	22.8 % vs. 29.6 %	25.8 % vs. 28.3 %	24.3 % vs. 30.3 %
Negative	69.7 % vs. 75.7 %	71.7 % vs. 74.2 %	77.2 % vs. 70.4 %	74.2 % vs. 71.7 %	75.7 % vs. 69.7 %
OR (95% CI)	1.352 (0.810-2.257)	1.134 (0.607-2.119)	0,702 (0.427-1.155)	1.1374 (0.607-2.119)	0,740 (0.443-1.235)
P-value	0.249	0.692	0.164	0.692	0.249
Distant metastasis					
Positive	2.8 % vs. 3.2 %	3.3 % vs. 3.0 %	3.2 % vs. 3.0 %	3.0 % vs. 3.3 %	3.2 % vs. 2.8 %
Negative	97.2 % vs. 96.8 %	96.7 % vs. 97.0 %	96.8 % vs. 97.0 %	97.0 % vs. 96.7 %	96.8 % vs. 97.2 %
OR (95% CI)	0.853 (0.216-3.366)	1.116 (0.231-5.395)	1.072 (0.304-3.775)	1.116 (0.231-5.95)	1.172 (0.297-4.624)
P-value	0.821	0.891	0.914	0.891	0.821
Grade					
G1 and G2	88.1 % vs. 88.4 %	87.7 % vs. 89.1 %	89.8 % vs. 86.9 %	89.1 % vs. 84.7 %	88.4 % vs. 88.1 %
G3	11.9 % vs. 11.6 %	15.3 % vs. 10.9 %	10.2 % vs. 13.1 %	10.9 % vs. 15.3 %	11.6 % vs. 11.9 %
OR (95% CI)	1.035 (0.507-2.112)	1.471 (0.656-3.299)	0.753 (0.380-1.493)	1.471 (0.656-3.299)	0.967 (0.473-1.973)
P-value	0.926	0.349	0.417	0.349	0.926

OR: Odds ratio; Cl: confidence intervals.

Table S2. Associations between *KRas* rs61764370 polymorphism and LSCC clinicopathological features.

Parameter	Genotype model			Allelic model	
	**TT vs. TG and GG**	**GG vs. TG and TT**	**TG vs. GG and TT**	**T carrier vs. T noncarrier**	**G carrier vs. G noncarrier**
Tumor stage					
T1 and T2	52.5 % vs. 60.5 %	0.0 % vs. 53.5 %	60.5 % vs. 52.5 %	53.5 % vs. 0.0 %	60.5 % vs. 52.5%
T3 and T4	47,5 % vs. 39.5 %	0.0 % vs. 46.5 %	39.5 % vs. 47.5 %	46.5 % vs. 0.0 %	39.5 % vs. 47.5 %
OR (95% CI)	1.386 (0.720-2.666)	-	0.722 (0.375-1.388)	-	0.722 (0.375-1.388)
P-value	0.328	-	0.328	-	0.328
Lymph node status					
Positive	63.7 % vs. 65.2 %	100 % vs. 63.8 %	61.9 % vs. 64.2 %	63.4 % vs. 100 %	62.4 % vs. 64.1 %
Negative	36.3 % vs. 36.8 %	0.0 % vs. 63.8 %	38.1 % vs. 35.8 %	36.6 % vs. 0.0 %	37.6 % vs. 35.9 %
OR (95% CI)	0.934 (0.589-1.482)	-	0.908 (0.567-1.456)	-	0,928 (0.580-1.485)
P-value	0.772	-	0.689	-	0.756
Distant metastasis					
Positive	2.5 % vs. 7.0 %	0.0 % vs. 3.1 %	7.0 % vs. 2.5 %	3.1 % vs. 0.0 %	7.0 % vs. 2.5 %
Negative	97.5 % vs. 93.0 %	0.0 % vs. 96.9 %	93.0 % vs. 97.5 %	96.9 % vs. 0.0 %	93.0 % vs. 97.5 %
OR (95% CI)	0.337 (0.084-1.356)	-	2.968 (0.737-11.946)	-	2.968 (0.737-11.946)
P-value	0.126	-	0.126	-	0.126
Grade					
G1 and G2	88.7 % vs. 86.0 %	0.0 % vs. 88.3 %	86.0 % vs. 14.0 %	88.3 % vs. 0.0 %	86.0 % vs. 88.7 %
G3	11.3 % vs. 14.0 %	0.0 % vs. 11.7 %	88.7 % vs. 11.3 %	11.7 % vs. 0.0 %	14.0 % vs. 11.3 %
OR (95% CI)	0.789 (0.309-2.016)	-	1.267 (0.496-3.236)	-	1.267 (0.496-3.236)
P-value	0.621	-	0.621	-	0.621

OR: Odds ratio; Cl: confidence intervals.

Table S3. Associations between *KRas* rs7973450 polymorphism and LSCC clinicopathological features.

**Parameter**	**Genotype model**			**Allelic model**	
	**TT vs. TG and GG**	**GG vs. TG and TT**	**TG vs. GG and TT**	**T carrier vs. T noncarrier**	**G carrier vs. G noncarrier**
Tumor stage					
T1 and T2	55.0 % vs. 51.5 %	50.0 % vs. 53.8 %	51.8 % vs. 54.5 %	53.8 % vs. 50.0 %	51.5 % vs. 55.0%
T3 and T4	45.5 % vs. 48.5 %	50.0 % vs. 46.2 %	48.2 % vs. 45.5 %	46.2 % vs. 50.0 %	48.5 % vs. 45.0 %
OR (95% CI)	0.869 (0.559-1.350)	1.163 (0.489-2.764)	1.115 (0.707-1.758)	0.860 (0.362-2.043)	1.151 (0.741-1.789)
P-value	0.531	0.732	0.640	0.732	0.531
Lymph node status					
Positive	63.4 % vs. 64.5 %	68.8 % vs. 63.5 %	62.5 % vs. 64.6 %	63.1 % vs. 72.7 %	63.6 % vs. 64.1 %
Negative	36.6 % vs. 35.5 %	31.2 % vs. 36.5 %	37.5 % vs. 35.4 %	36.9 % vs. 27.3 %	36.4 % vs. 35.9 %
OR (95% CI)	0.953 (0.691-1.313)	1.265 (0.673-2.380)	0.15 (0.656-1.277)	0,640 (0.346-1.186)	0,981 (0.711-1.352)
P-value	0.768	0.466	0.601	0.156	0.906
Distant metastasis					
Positive	2.1 % vs. 4.4 %	0.0 % vs. 3.1 %	4.4 % vs. 2.3 %	3.0 % vs. 4.5 %	4.4 % vs. 2.1 %
Negative	97.9 % vs. 95.6 %	0.0 % vs. 96.9 %	95.6 % vs. 97.7 %	97.0 % vs. 95.5 %	95.6 % vs. 97.9 %
OR (95% CI)	0.463 (0.128-1.675)	1.589 (0.189-12.955)	1.908 (0.541-6.735)	0.639 (0.077-5.282)	2.158 (0.597-7.798)
P-value	0.241	0.677	0.315	0.677	0.241
Grade					
G1 and G2	89.5 % vs. 86.6 %	81.0 % vs. 88.8 %	87.6 % vs. 12.4 %	88.8 % vs. 81.0 %	86.6 % vs. 89.5 %
G3	10.5 % vs. 13.4 %	19.0 % vs. 11.2	12.4 % vs. 11.3 %	11.2 % vs. 19.0 %	13.4 % vs. 10.5 %
OR (95% CI)	0.754 (0.382-1.485)	1.869 (1.594-5.878)	1.108 (0.549-2.237)	0.535 (0.170-1.684)	1.327 (0.673-2.616)
P-value	0.414	0.285	0.775	0.285	0.414

OR: Odds ratio; Cl: confidence intervals.

Table S4. Associations between *NRas* rs14804 polymorphism and clinicopathological features

**Parameter**	**Genotype model**			**Allelic model**	
	**TT vs. CT and CC**	**CC vs. CT and TT**	**CT vs. CC and TT**	**T carrier vs. T noncarrier**	**C carrier vs. C noncarrier**
Tumor stage					
T1 and T2	49.5 % vs. 58.7 %	**83.3 % vs. 51.8 %**[1a]	55.2 % vs. 52.5 %	**51.8 % vs. 83.3 %**[1b]	58.7 % vs. 49.5 %
T3 and T4	50.5 % vs. 41.3 %	**16.7 % vs. 48.2 %**	44.8 % vs. 47.5 %	**48.2 % vs. 16.7 %**	41.3 % vs. 50.5 %
OR (95% CI)	1.455 (0.936-2.261)	0.215 (0.061-0.757)	0.896 (0.573-1.402)	4.656 (1.321-16.407)	0.687 (0.442-1.068)
P-value	0.095	**0.017**[1]	0.631	**0.017**[1]	0.095
Lymph node status					
Positive	61.2 % vs. 66.8 %	72.3 % vs. 63.2 %	64.9 % vs. 63.2 %	**62.8 % vs. 32.3 %**[1c]	66.0 % vs. 61.2 %
Negative	38.8 % vs. 33.2 %	27.3 % vs. 36.8 %	35.1 % vs. 36.8 %	**37.2 % vs. 67.7 %**	34.0 % vs. 38.8 %
OR (95% CI)	0.785 (0.572-1.079)	1.521 (0.787-2.942)	1.078 (0.780-1.489)	3.646 (2.334-9.249)	1.232 (0.896-1.694)
P-value	0.135	0.213	0.651	**0.023**[1]	0.200
Distant metastasis					
Positive	3.3 % vs. 2.8 %	0.0 % vs. 3.2 %	3.2 % vs. 3.0 %	3.2 % vs. 0.0 %	2.8 % vs. 3.3 %
Negative	96.7 % vs. 97.2 %	100 % vs. 96.8 %	96.8 % vs. 97.0 %	96.8 % vs. 100 %	97.2 % vs. 96.7 %
OR (95% CI)	1.171 (0.324-4.232)	-	1.080 (0.299-3.905)	-	0.854 (0.236-3.084)
P-value	0.809	-	0.907	-	0.809
Grade					
G1 and G2	89.6 % vs. 86.6 %	83.3 % vs. 88.6 %	87.1 % vs. 89.1 %	88.8 % vs. 83.3 %	86.6 % vs. 89.6 %
G3	10.4 % vs. 13.4 %	16.7 % vs. 11.4 %	12.9 % vs. 10.9 %	11.4 % vs. 16.7 %	13.4 % vs. 10.4 %
OR (95% CI)	0.750 (0.381-1.477)	1.554 (0.428-5.638)	01.205 (0.607-2.396)	0.502 (0.177-2.334)	1.333 (0.677-2.625)
P-value	0.405	0.502	0.594	0.502	0.405

[1]Significant; [a]Fisher's exact test; P=0.009; [b]Fisher's exact test, P=0.013; [c]Fisher's exact test, P=0,0012 OR: Odds ratio; Cl: confidence intervals.

Table S5. Associations between *MAPK1* rs9340 polymorphism and LSSC clinicopathological features

**Parameter**	**Genotype model**			**Allelic model**	
	**CC vs. CT and TT**	**TT vs. CT and CC**	**CT vs. CC and TT**	**C carrier vs. C noncarrier**	**T carrier vs. T noncarrier**
Tumor stage					
T1 and T2	54.1 % vs. 53.2 %	57.4 % vs. 52.7 %	51.8 % vs. 55.2 %	52.7 % vs. 57.4 %	53.2 % vs. 54.1 %
T3 and T4	45.9 % vs. 46.8 %	42.6 % vs. 47.3 %	48.2 % vs. 44.8 %	47.3 % vs. 42.6 %	46.8 % vs. 45.9 %
OR (95% CI)	1.964 (0.608-1.529)	0.828 (0.459-1.493)	1.146 (0.742-1.770)	1.207 (0.670-2.177)	1.038 (0.654-1.646)
P-value	0.875	0.531	0.539	0.531	0.875
Lymph node status					
Positive	62.1 % vs. 64.7 %	60.9 % vs. 64.5 %	65.3 % vs. 62.5 %	64.0 % vs. 60.9 %	64.1 % vs. 62.1 %
Negative	37.9 % vs. 35.3 %	39.1 % vs. 35.5 %	34.7 % vs. 37.5 %	36.0 % vs. 39.1 %	35.9 % vs. 37.9 %
OR (95% CI)	0.894 (0.637-1.253)	0.856 (0.567-1.294)	1.129 (0.823-1.549)	1.145 (0.757-1.731)	1.092 (0.779-1.532)
P-value	0.515	0.462	0.451	0.522	0.609
Distant metastasis					
Positive	0.9 % vs. 4,1 %	**14.8 % vs. 0.7 %**[1a]	4.9 % vs. 1.2 %	3.3 % vs. 1.9 %	4.1 % vs. 0.9 %
Negative	99.1 % vs. 95.9 %	**85.2 % vs. 99.3 %**	95.1 % vs. 98.8 %	96.7 % vs. 98.1 %	95.9 % vs. 99.1 %
OR (95% CI)	0.215 (0.027-1.719)	4.553 (1.069-8.461)	4.128 (0.863-19.746)	1.807 (0.224-14.563)	4.651 (0.582-37.189)
P-value	0.147	**0.041**[1]	0.156	0.579	0.147
Grade					
G1 and G2	85.3 % vs. 89.8 %	88.7 % vs. 88.2 %	90.2 % vs. 86.4 %	88.2 % vs. 88.7 %	89.8 % vs. 85.3 %
G3	14.7 % vs. 10.2 %	11.3 % vs. 11.8 %	9.8 % vs. 13.6 %	11.8 % vs. 11.3 %	10.2 % vs. 14.7 %
OR (95% CI)	1.517 (0.761-3.024)	0.957 (0.379-2.418)	0.693 (0.349-1.373)	1.044 (0.414-2.637)	0.659 (0.331-1.314)
P-value	0.236	0.927	0.693	0.927	0.236

*Significant; [a]Fisher's exact test, P=0.005; OR: Odds ratio; Cl: confidence intervals.

Table S6. Association between *KRas* rs712 and rs7973450 polymorphisms in haplotype models and LSCC clinicopathologic features

Parameter	Haplotype model		
	**TG haplotype carriers *vs.* TG haplotype noncarriers**	**GT haplotype carriers *vs.* GT haplotype noncarriers**	**TT haplotype carriers *vs.* TT haplotype noncarriers**
Tumor stage			
T1 and T2	51.5 % *vs.* 54.9 %	54.9 % *vs.* 47.5 %	54.6 % *vs.* 51.9 %
T3 and T4	48.5 % *vs.* 45.1 %	45.1 % *vs.* 52.5 %	45.4 % *vs.* 48.1 %
OR (95% CI)	1.144 (0.735-1.782)	1.343 (0.769-2.345)	0.898 (0.576-1.399)
P-value	0.551	0.300	0.634
Lymph node status			
Positive	**45.8 % *vs.* 26.7 %**[1a]	25.9 % *vs.* 27.9 %	23.5 % *vs.* 30.5 %
Negative	**54.2 % *vs.* 73.3 %**	72.1 % *vs.* 72.1 %	76.5 % *vs.* 69.5 %
OR (95% CI)	3.954 (2.577-6.578)	1.103 (0.591-2.057)	0.698 (0.424-1.147)
P-value	**0.015**[1]	0.758	0.156
Distant metastasis			
Positive	4.5 % *vs.* 2.1 %	3.0 % *vs.* 3.3 %	3.1 % *vs.* 3.1 %
Negative	95.5 % *vs.* 97.9 %	97.0 % *vs.* 96.7 %	96.9 % *vs.* 96.9 %
OR (95% CI)	2.274 (0.629-8.219)	1.093 (0.226-5.282)	1.003 (0.277-3.624)
P-value	0.210	0.912	0.997
Grade			
G1 and G2	87.7 % *vs.* 88.7 %	89.1 % *vs.* 85.0 %	89.2 % *vs.* 86.9 %
G3	12.3 % *vs.* 11.3 %	10.9 % *vs.* 15.0 %	10.8 % *vs.* 13.1 %
OR (95% CI)	1.104 (0.556-2.192)	1.436 (0.641-3.218)	0.802 (0.406-1.586)
P-value	0.778	0.379	0.526

[1]Significant; [a]Fisher's exact test, P=0.025; OR: Odds ratio; Cl: confidence intervals.
